# Early and Long-Term Outcomes of Surgical Treatment of Ebstein’s Anomaly

**DOI:** 10.21470/1678-9741-2018-0333

**Published:** 2019

**Authors:** Guilherme Viotto Rodrigues da Silva, Leonardo Augusto Miana, Luiz Fernando Caneo, Aída Luiza Ribeiro Turquetto, Carla Tanamati, Juliano G. Penha, Fabio B Jatene, Marcelo B Jatene

**Affiliations:** 1 Instituto do Coração do Hospital das Clínicas da Faculdade de Medicina da Universidade de São Paulo (InCor-HCFMUSP), São Paulo, SP, Brazil.

**Keywords:** Ebstein Anomaly, Tricuspid Valve Insufficiency, Ventricular Dysfunction, Right, Cardiac Surgical Procedures, Survival Rate

## Abstract

**Objective:**

This study aimed to evaluate Ebstein’s anomaly surgical correction and its early and long-term outcomes.

**Methods:**

A retrospective analysis of 62 consecutive patients who underwent surgical repair of Ebstein’s anomaly in our institution from January 2000 to July 2016. The following long-term outcomes were evaluated: survival, reoperations, tricuspid regurgitation, and postoperative right ventricular dysfunction.

**Results:**

Valve repair was performed in 46 (74.2%) patients - 12 of them using the Da Silva cone reconstruction; tricuspid valve replacement was performed in 11 (17.7%) patients; univentricular palliation in one (1.6%) patient; and the one and a half ventricle repair in four (6.5%) patients. The patients’ mean age at the time of surgery was 20.5±14.9 years, and 46.8% of them were male. The mean follow-up time was 8.8±6 years. The 30-day mortality rate was 8.06% and the one and 10-year survival rates were 91.9% both. Eleven (17.7%) of the 62 patients required late reoperation due to tricuspid regurgitation, in an average time of 7.1±4.9 years after the first procedure.

**Conclusion:**

In our experience, the long-term results of the surgical treatment of Ebstein's anomaly demonstrate an acceptable survival rate and a low incidence of reinterventions.

**Table:** 

Abbreviations, acronyms & symbols			
ASD	= Atrial septal defect	PV	= Pulmonary valve
BDG	= Bidirectional Glenn	RA	= Right atrial
BK	= Bundle of Kent	RV	= Right ventricle/ventricular
CABG	= Coronary artery bypass graft	SD	= Standard deviation
CR	= Cone reconstruction	SPSS	= Statistical Package for the Social Sciences
EA	= Ebstein’s anomaly	TR	= Tricuspid regurgitation
ICU	= Intensive care unit	TV	= Tricuspid valve
IQR	= Interquartile range	TVR	= Tricuspid valve replacement
LVEF	= Left ventricular ejection fraction	VRP	= Non-Cone valve repair
PAPVC	= Partial anomalous pulmonary venous connection	VSD	= Ventricular septal defect
PDA	= Patent ductus arteriosus	WPW	= Wolff-Parkinson-White

## INTRODUCTION

In 1866, Wilhelm Ebstein described the anatomical findings related to the heart of a 19-year-old man with cyanosis, palpitations, and dyspnea. The postmortem findings were tricuspid valve (TV) anomaly with dilation of the right ventricle (RV) and patent foramen ovale. Ebstein’s anomaly (EA) is a primary disorder of TV and accounts for approximately 1% of congenital heart defects^[1,2]^.

The EA is characterized by various degrees of adherence and displacement of the septal and posterior leaflet into the RV, resulting in a rotational and apical displacement of TV, an abnormal atrialized portion of RV, tricuspid regurgitation (TR), and arrhythmias, producing several anatomical variations^[3]^.

Surgical treatment of EA was firstly reported by Hunter and Lillehei in 1958^[4]^. Numerous techniques of tricuspid repair have been published by several authors in an attempt to eliminate TR and restore RV geometry. However, high incidence of tricuspid dysfunction and tricuspid valve replacement (TVR) was observed^[5-7]^.

In 2004, Da Silva et al.^[8]^ proposed the cone reconstruction (CR) of TV, shifting the paradigm of EA surgical management. Although this technique uses some principles of the Carpentier concepts, bringing the TV leaflets to the true tricuspid annulus level and longitudinal plication of the atrialized RV, it adds the advantage of leaflet to leaflet coaptation and restores RV geometry and function avoiding a prosthetic ring, thus enabling growth and flexibility of the tricuspid annulus^[8]^.

We evaluated the outcomes of patients who underwent surgical correction of EA in our single center.

## METHODS

We carried out a retrospective, single-center analysis study of all consecutive patients who underwent surgical treatment for EA. Inclusion criteria were the diagnosis of EA + atrioventricular and ventriculoarterial concordance. Exclusion criteria were patients with complex conotruncal abnormalities and neonatal EA presentation.

Indications for operations were congestive heart failure symptoms, increasing cyanosis, atrial or ventricular arrhythmias not amenable to another therapy, deteriorating systolic function, or progressive dilatation of RV.

Operative management of EA could be either a biventricular repair, one and a half ventricle repair, or univentricular palliation. One and a half repair was performed when the RV was judged not capable of supporting the pulmonary circulation (cases of severe EA and/or impaired RV function). Univentricular palliation was reserved for cases of severe RV hypoplasia. Reconstruction of TV or TVR, selective plication of the atrialized RV, and correction of any associated anomaly was also performed. Until 2010, the techniques of TV repair used in our institution were those reported by Danielson et al.^[6]^ and Carpentier et al.^[7]^, and those consisted of a monocusp repair at the level of the functional annulus. After 2010, we started to perform the Da Silva CR reported by Da Silva et al.^[8]^ in all cases of EA repair, achieving improvement in TV regurgitation and in RV geometry and function.

Echocardiography was routinely performed preoperatively, intraoperatively, and during follow-up. TR was categorized into four groups: none, mild, moderate, and severe.

The institutional ethics committee on human research approved this study. Due to the retrospective nature of the study, the need for individual patient consent was waived.

Data on demographic variables, intraoperative procedures, and postoperative outcomes were collected retrospectively. Data were expressed as absolute and percentage frequency values. For variables with homogeneous distribution, parametric tests were performed, and the results were presented in mean and standard deviation. As for the variables with non-homogeneous distribution, we performed non-parametric tests, and the results were presented in median and interquartile range (IQR). The normality test used was the Kolmogorov-Smirnov test. Freedom from reoperation and cumulative survival rates were analyzed according to Kaplan-Meier test. We considered as statistically significant differences the results with values of *P*<0.05. Statistical analysis was conducted using the IBM Statistical Package for the Social Sciences (SPSS) Statistics^®^ 20 for Windows (Chicago, Illinois, United States).

## RESULTS

From January 2000 through July 2016, 62 consecutive patients were submitted to surgical correction of EA in our institution. The patients’ mean age at time of surgery was 20.5±14.9 years (7 months-68.8 years), and 46.8% of them were male. One patient had one previous cardiac procedure (atrial septal defect closure) to the EA correction.

Preoperative transthoracic echocardiography demonstrated severe TR in 55 (87.5%) patients, impaired RV systolic function in 16 (25.8%) patients, and low ejection fraction of left ventricle in two (3.2%) patients. Forty-one patients had an associated congenital heart lesion, and arrhythmias were present in 20 (32.3%) patients. Demographic and preoperative data are shown in Table 1.

**Table 1:** Patients’ demographic and preoperative data.

Variables	Total, n=62 (%)
Male	29 (46.8%)
At presentation	
Mean age & #x00b1; SD (years)	20.5±14.9
Weight (kg)	45.1±3.4
Prior cardiac surgery	1 (1.61%)
Type of associated anomaly	
ASD	40 (64.5%)
VSD	3 (4.8%)
Severe PV anomaly	5 (8.1%)
PDA	1 (1.61%)
PAPVC	1 (1.61%)
Severity of TR	
Mild	1 (1.6%)
Moderate	6 (9.7%)
Severe	55 (88.7%)
Severity of RV systolic dysfunction	
Mild	7 (11.3%)
Moderate	5 (8.1%)
Severe	4 (6.5%)
Low LVEF	2 (3.2%)
Arrhythmias	20 (32.3%)
WPW syndrome	7 (11.3%)
Supraventricular tachycardia	6 (9.7%)
Atrial flutter	5 (8.1%)
Chronic atrial fibrillation	2 (3.2%)
Previous ablation	9 (14.5%)

ASD=atrial septal defect; kg=kilograms; LVEF=left ventricular ejection fraction; PAPVC=partial anomalous pulmonary venous connection; PDA=patent ductus arteriosus; PV=pulmonary valve; N=number; RV=right ventricular; SD=standard deviation; TR=tricuspid regurgitation; VSD=ventricular septal defect; WPW=Wolff-Parkinson-White

Non-cone valve repair (VRP) was performed in 34 (54.8%) patients; Da Silva CR in 12 (19.4%) patients; TVR in 11 (17.7%) patients - all the replacements were performed with biological prosthesis; univentricular palliation in one (1.6%) patient, and one and half ventricle repair in four (6.5%) patients. The mean cardiopulmonary bypass time was 160.6±61.1 minutes with a mean aortic cross-clamp time of 110.3±35.4 minutes. The median length of stay in the intensive care unit was six (IQR 3-15,5) days, and the median hospital length of stay after the procedure was nine (IQR 7-18) days. Operative data are shown in Table 2.

**Table 2:** Patients’ operative data.

Variables	Total, n=62 (%)
VRP	34 (54.8%)
+CABG	2 (3.2%)
+RA maze	1 (1.6%)
CR	12 (19.4%)
+RA maze	1 (1.6%)
+VSD repair	1 (1.6%)
+PV procedure	2 (3.2%)
TVR	11 (17.7%)
+RA maze	4 (6.5%)
+VSD repair	1 (1.6%)
+PV procedure	3 (4.8%)
+Surgical division BK	1 (1.6%)
Univentricular palliation	1 (1.6%)
One and a half ventricle repair	4 (6.5%)
TVR + BDG	2 (3.2%)
CR + BDG	1 (1.6%)
CR + BDG+ VSD + PDA repair	1 (1.6%)
Bypass time, min	160.6±61.1
Cross-clamp time, min	110.3 ± 35.4
Length of ICU stay, days	6 (IQR 3-15.5)
Length of hospital stay after procedure, days	9 (IQR 7-18)
Delayed sternal closure	3 (4.8%)
Centrifugal pump	2 (3.2%)

BDG=bidirectional Glenn; BK=bundle of Kent; CABG=coronary artery bypass graft; CR=cone reconstruction; ICU=intensive care unit; IQR=interquartile range;min=minutes;  N=number; PDA=patent ductus arteriosus; PV=pulmonary valve; RA=right atrial; TVR=tricuspid valve replacement; VRP=non-cone valve repair; VSD=ventricular septal defect

There were five deaths, all in the first 30 postoperative days (one - VRP; two - TVR; two - one and half ventricle repair). There were no late deaths. The causes of death were three multi-organ dysfunctions + septic sepsis and two cardiogenic shocks. Centrifugal pump was required in two patients (one patient who underwent TVR and survived and one who underwent one and a half ventricle repair and died).

Early reoperation (during the hospital stay) occurred in two patients (3.2%). One patient (VRP) for recurrent TR, who underwent valve replacement, and one patient (TVR) who required centrifugal pump, was weaned off from the device, and underwent a bidirectional Glenn procedure - this patient is currently listed for heart transplantation due to biventricular dysfunction. Two patients required pacemaker implantation because they presented advanced heart block during hospitalization, both underwent VRP.

Postoperative infection was observed in four patients (6.5%), in a mean of 4.5±0.5 postoperative days; the most common site of infection was the lung, followed by wound infection.

Serial echocardiograms were performed in the follow-up period to evaluate TR and RV systolic function. There were no significant differences on the echocardiographic data when assessing the entire cohort. Clinically important TV stenosis was not observed in the VRP and CR groups. Twenty-six patients who underwent VRP and eight patients who underwent CR had moderate or severe TV insufficiency (*P*=0.511). Late qualitative assessment of RV dysfunction showed no statistical difference from the preoperative baseline. Bioprosthesis dysfunction (stenosis and/or insufficiency greater than moderate) was observed in six patients who underwent TVR. Postoperative data are shown in Table 3.

**Table 3:** Patients’ postoperative data.

Variables	VRP (n)	CR (n)	TVR (n)	*P*
TV regurgitation grade				0.511^a^*
None/mild	7	4		
Moderate	5	3		
Severe	21	5		
Bioprosthesis dysfunction			6	
RV dysfunction grade				0.181^a^**
None/mild	22	11	4	
Moderate	6	0	3	
Severe	4	1	3	
Early reoperation	1	0	1	0.627^a^**
Late reoperation	8	2	2	0.170^a^**

a Chi-square test;

* between VRP and CR group;

** between all groups

CR=cone reconstruction; n=number; RV=right ventricular; TV=tricuspid valve; TVR=tricuspid valve replacement; VRP=non-cone valve repair

**Fig. 1:**
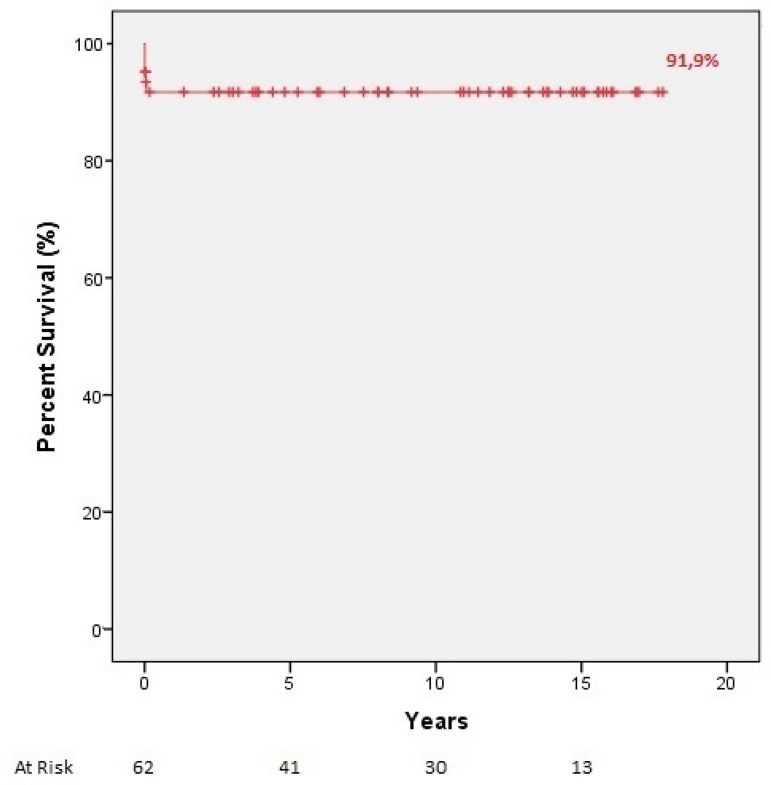
Late survival (Kaplan-Meier) for entire cohort after surgical treatment of Ebstein’s anomaly (n=62).

Mean follow-up time was 8.8±6 years (range: 1 day - 17.8 years). There were no late deaths, and the overall survival rate at one, five, and 10 years were all 91.9% (Figure 1). Eleven (17.7%) of 62 patients required late reoperation due TR, in an average time of 7.1±4.9 years after the first procedure. Three patients required a second reoperation - two patients because of bioprosthesis dysfunction and one patient due to TV regurgitation - in an average time of 6.3±4.7 years after the first reoperation (Table 4). Freedom from late reoperation was 79% at 15 years.

**Table 4:** Characteristics of the patients who underwent late reoperation due to tricuspid regurgitation.

Patient	FP	Reop.	Years since FP	Sec. reop.	Years since first reop.
1	VRP	TVR	16.3		
2	VRP	TVR	9.1		
3	VRP	TVR	1.1	TVR	10.2
4	VRP	TVR	11.3		
5	VRP	TVR	6.4	TVR	7.6
6	VRP	TVR	12.4		
7	VRP	VRP	3.4		
8	TVR	TVR	7.7		
9	VRP	Annuloplasty	1.3	TVR	1.0
10	CR	Plasty	7.8		
11	CR	Plasty	2.1		
Mean±SD			7.1±4.9		6.3±4.7

CR=cone reconstruction; FP=first procedure; Reop.=procedure performed in the reoperation; SD=standard deviation; Sec. reop.=procedure performed in the second reoperation; TVR=tricuspid valve replacement; VRP=non-cone valve repair

## DISCUSSION

The ideal repair of TV and RV is the anatomical repair, and the surgical treatment of EA remains a challenge. The mechanism of TR is related to restrictive leaflet movements, and the first surgical techniques were focused on TV monocusp repair. In 1988, Carpentier et al.^[7]^ described a new technique that consisted of longitudinal plication of the RV and return of the TV to the correct level, reinforced with a prosthetic ring. Danielson et al.^[6]^ proposed a transverse plication of the atrialized RV, posterior tricuspid annuloplasty, and right reduction atrioplasty. Quaegebeur et al.^[9]^ modified the technique proposed by Carpentier, without the use of a prosthetic ring. All these techniques reported a high incidence of TV dysfunction, and TVR was necessary. Until 2010, the methods of TV repair used in our institution were those published by Danielson et al. and Carpentier et al. and we found a high incidence of TR.

In 2004, Da Silva et al.^[8]^ reported a new encouraging surgical technique in which the main feature was CR of the TV. This technique uses some principles of the Carpentier concepts, bringing the TV leaflets to the true tricuspid annulus level and longitudinal plication of the atrialized RV, that mimics the normal TV anatomy. In addition to eliminating TR, CR restores the RV geometry and function, without a prosthetic ring, thus enabling growth of the tricuspid annulus^[10]^. Late outcomes were reported by Da Silva et al. with low mortality and efficient TV performance^[11,12]^.

After 2010, our group started to perform the CR reported by Da Silva et al.^[8]^ in all cases of TV repair, and we observed good RV function and low incidence of severe TR in long-term follow-up. Two patients who underwent CR required late reoperation due TR, and TV re-repair was successful in all cases. In a recent study of 235 patients reported by the Mayo Clinic group, CR proved to be safe and effective, with a reduction in TR and changes in RV remodeling^[13]^. Encouraging outcomes were reported by other studies performing CR^[14,15]^. Beroukhim et al.^[16]^ also reported improvements in RV volume and TR, besides improvement in left ventricular systolic function and synchrony in patients who underwent CR^[17]^.

Symptomatic EA in early infancy and newborns may present with cyanosis and severe congestive heart failure. In our study, we excluded the newborn patients because they mainly represent the worse spectrum of this disease, and the biventricular repair is often not applied. Besides, outcomes in newborn EA are poorer when compared with the overall EA population^[18-20]^.

In our experience, freedom from late reoperation was 79% in 15 years. Twelve (19.4%) patients required late reoperation in an average time of 5.8 ± 5.2 years after the first procedure and three patients required a second reoperation. A limitation of this study is the small follow-up time to evaluate the need for reoperation in all TVR patients due the durability of the bioprosthesis. Similar results were reported by Luu et al.^[21]^ in a study of 51 patients with EA, and 18% of them required tricuspid reoperation during their 21-year follow-up. Other studies reported freedom from reoperation ranging from 88.7% to 92.9% in 10 and 20-year follow-ups, respectively^[22,23]^.

Total mortality in the current study was 8.1% for all patients in the first 30 postoperative days, and there were no late deaths. In a cohort of 539 patients, Brown et al.^[24]^ reported total mortality of 29% in a 20-year follow-up and the 30-day and one-, five-, 10-, 15-, and 20-year survival rates were 94%, 92%, 88%, 85%, 81%, and 71%, respectively. Our overall survival rate at one, five, and 10 years was 91.9%, respectively.

## CONCLUSION

In conclusion, surgical management of EA remains a challenge. In this series of patients, our long-term outcomes demonstrate an acceptable survival rate, mortality limited to the immediate postsurgical period, and a low incidence of reinterventions and morbidity.

**Table:** 

Authors’ roles & responsibilities	
GVRS	Substantial contributions to the conception or design of the work; or the acquisition, analysis, or interpretation of data for the work; drafting the work or revising it critically for important intellectual content; final approval of the version to be published
LAM	Substantial contributions to the conception or design of the work; or the acquisition, analysis, or interpretation of data for the work; drafting the work or revising it critically for important intellectual content; final approval of the version to be published
LFC	Substantial contributions to the conception or design of the work; or the acquisition, analysis, or interpretation of data for the work; drafting the work or revising it critically for important intellectual content; final approval of the version to be published
ALRT	Drafting the work or revising it critically for important intellectual content; final approval of the version to be published
CT	Drafting the work or revising it critically for important intellectual content; final approval of the version to be published
JGP	Drafting the work or revising it critically for important intellectual content; final approval of the version to be published
FBJ	Final approval of the version to be published
MBJ	Final approval of the version to be published
